# Patients with Restless Leg Syndrome Have Lower Coronary Flow Velocity Reserve Compared to Healthy Controls: Case–Control Study

**DOI:** 10.3390/jcdd13060270

**Published:** 2026-06-15

**Authors:** Göksel Güz, Rasim Onur Karaoğlu, Sezen Kumaş Solak, Serdar Demirgan

**Affiliations:** 1 Department of Cardiology, Medicana International Hospital, 34520 Istanbul, Turkey; 2 Department of Anesthesiology and Reanimation, Bagcılar Training Research Hospital, University of Health Sciences, 34200 Istanbul, Turkey; rasimonur.karaoglu@sbu.edu.tr (R.O.K.); sezenkumassolak@gmail.com (S.K.S.); serdar.demirgan@sbu.edu.tr (S.D.)

**Keywords:** coronary flow velocity reserve, coronary heart disease, endothelial dysfunction, restless leg syndrome

## Abstract

Objective: Restless leg syndrome (RLS) has been associated with an increased risk of vascular disorders, which suggests that endothelial dysfunction plays an important role in the pathogenesis of RLS. In this study, we aimed to evaluate coronary endothelial dysfunction in RLS patients using coronary flow velocity reserve (CFVR) and compared it with healthy controls. Methodology: In this study, the participants were divided into two groups as group RLS (*n* = 42) and group HC (*n* = 41). The primary outcome was the CFVR compared between groups. The number of participants with a CFVR value below 2.0 was also evaluated. In addition, a correlation between the international restless legs scale (IRLS) and CFVR, white-blood-cell-count (WBC), and C-reactive protein (CRP) was analyzed. Secondary outcomes were the WBC, hemoglobin, CRP, blood glucose, high-density lipoprotein (HDL), low-density lipoprotein (LDL), and creatinine compared between the two groups. Results: In the group RLS, CFVR was measured lower than healthy controls (*p* < 0.001). When the groups were compared in terms of the number of participants with a CFVR less than 2.0, the difference between the groups was significant (*p* < 0.001, 0/41 in group HC and 14/42 in group RLS). Patients with RLS had higher WBC and CRP values. There was a negative correlation between CFVR and IRLS (*p* < 0.001). The relationship between WBC, CRP, and IRLS was not statistically significant (*p* = 0.691). Conclusions: In this exploratory study, RLS patients had lower CFVR compared with healthy controls and a negative correlation was observed between RLS severity and CFVR. These findings warrant confirmation in larger, prospectively designed studies with multivariable adjustment. Therefore, we think that it may be beneficial to follow-up patients with RLS in terms of coronary heart disease. Clinical trial number: not applicable.

## 1. Background

Restless legs syndrome (RLS) is a chronic sensorimotor disorder characterized by an urge to move the legs accompanied by uncomfortable sensations, typically occurring during periods of rest [1]. Although traditionally considered a neurological condition, growing evidence suggests that RLS may also be associated with systemic vascular abnormalities and increased cardiovascular risk, including coronary heart disease and cerebrovascular events. Previous studies have reported a relationship between RLS and increased cardiovascular risk, including coronary heart disease [2].

Endothelial dysfunction is a key mechanism in the development of atherosclerosis and plays a central role in coronary heart disease [3]. In addition, epidemiological studies have demonstrated associations between sleep-related disorders and cardiovascular outcomes such as stroke and cardiac events [4]. However, methodological limitations in observational studies and conflicting findings across different cohorts make this relationship complex and not fully established [5,6]. Coronary flow velocity reserve (CFVR) is a non-invasive parameter that reflects coronary microvascular and endothelial function. It can detect early coronary vascular impairment even in the absence of significant epicardial coronary artery disease. Therefore, CFVR may provide a more direct evaluation of coronary endothelial function in patients with RLS.

In this study, we aimed to assess coronary endothelial function in patients with RLS using CFVR and to compare these findings with those of healthy controls.

## 2. Methods

Between October 2022 and February 2023, 702 patients with normal coronary arteries who underwent coronary angiography in our hospital were included in this cross-sectional observational study, and 58 of these were diagnosed with RLS for the first time (Group RLS). Participants were selected from patients undergoing coronary angiography for clinical indications and found to have normal coronary arteries. Diagnosis of RLS was established using the International Restless Legs Syndrome Study Group rating scale (IRLS), a previously validated and widely used instrument, to assess disease severity [7]. A ten-question scale was developed on the basis of repeated expert evaluation of potential items. Each question had a set of five response options graded from no RLS or impact (score = 0) to very severe RLS or impact (score = 4). This produced a total scale whose overall score could range from 0 to 40. Patients were classified into four groups with scores of mild (0–10), moderate (11–20), severe (21–30), and very severe (31–40) RLS according to this scale. Age and sex-matched healthy controls *(n* = 58) were selected from patients with normal coronary arteries with similar age and gender distribution to the RLS patients (Group HC). The exclusion criteria for participants were as follows: participants who use drugs that affect the CFVR, comorbidities likely to be associated with secondary RLS (such as pregnancy, chronic kidney disease, iron deficiency, or peripheral neuropathy), clinical conditions that could mimic RLS symptoms, and uncontrolled hypertension, diabetes mellitus, and dyslipidemia.

During the study period, all patients undergoing coronary angiography were routinely screened for COVID-19 using PCR testing, and only patients with negative PCR results were included in the study.

The study was approved by the ethics committee of Medicana International Hospital (037-24.06). Written informed consent was obtained from all participants at the time of clinical evaluation. Participants were categorized into two groups: the RLS group (*n* = 42) and the healthy control group (*n* = 41). The patients’ background characteristics (such as age, gender, body mass index (BMI), systolic arterial pressure (SAP), diastolic arterial pressure (DAP), and heart rate (HR)) were recorded and compared between groups. Information on clinical variables including comorbidities, medication use, smoking status and relevant laboratory parameters was recorded for all participants. The blood pressure and HR of the participants were measured just before the coronary angiography procedure. White blood cell count (WBC), hemoglobin, C-reactive protein (CRP), blood glucose, high-density lipoprotein cholesterol (HDL), low-density lipoprotein cholesterol (LDL), and serum creatinine values of the participants were also recorded.

**Figure 1:**
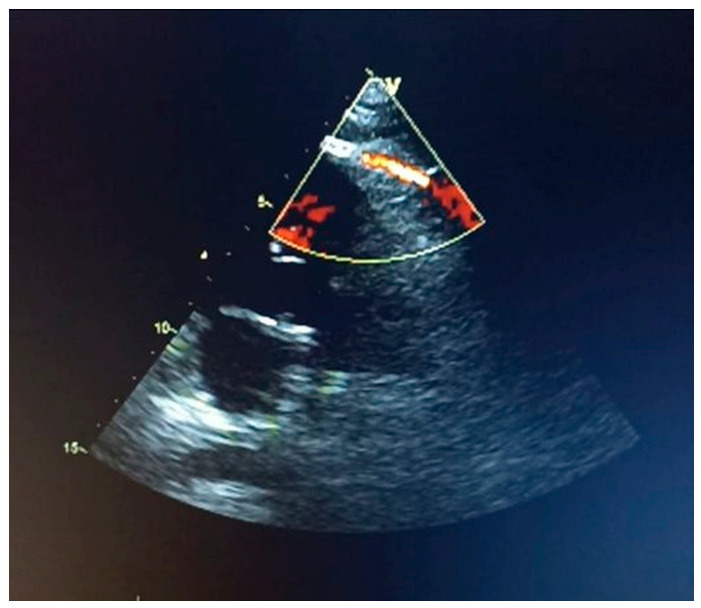
Transthoracic Doppler echocardiography. Color Doppler visualization of mid-diastolic LAD.

CFVR measurement was performed as previously described [8] using a Philips Affinity ultrasound system (Philips Healthcare, Amsterdam, The Netherlands). Hyperemia was induced pharmacologically using intravenous dipyridamole (Persantine^®^, Boehringer Ingelheim Pharmaceuticals, Inc., Ridgefield, CT, USA) (0.84 mg/kg) in accordance with standard stress echocardiography protocols. Average diastolic peak flow velocity (ADPV) was measured at baseline (ADPV_B_) and under hyperemic conditions (ADPV_H_) from the left anterior descending (LAD) coronary artery (Figure 1). The coronary flow velocity reserve was defined as the ratio of ADPV_H_ to ADPV_B_. A CFVR value < 2.0 was considered abnormal. All CFVR measurements were performed by an experienced cardiologist who was blinded to the clinical and laboratory data of the participants, including group allocation (RLS vs. healthy control). Inter- and intra-observer variability were not assessed in this study. All measurements were performed by a single experienced operator using a standardized protocol, which may have reduced inter-operator variability despite the absence of formal reproducibility analysis.

### 2.1. Primary and Secondary Outcomes

The primary outcome was the CFVR compared between group RLS and group HC. In addition, a correlation between IRLS and CFVR, WBC, and CRP values was analyzed. The number of participants with a CFVR value below 2.0 was also compared between groups. Secondary outcomes were the WBC, hemoglobin, CRP, blood glucose, HDL, LDL, and serum creatinine values compared between the two groups.

### 2.2. Statistical Analyses

Quantitative data were summarized as mean ± standard deviation along with median (minimum–maximum), whereas frequency and percentage were used for qualitative data. The Shapiro–Wilk test was used to check if a quantitative variable follows a normal distribution. Demographic and clinical characteristics between healthy controls and patients with RLS were compared using Student’s *t*-test, Mann–Whitney U test, and chi-square test, accordingly. Correlation between IRLS and CFVR, WBC, and CRP was assessed with Kendall’s tau-b (τb) correlation coefficient. The area under the receiver operating characteristic (ROC) curve (AUC) along with its 95% confidence interval (CI) was given to assess the diagnostic value of CFVR, WBC, and CRP to discriminate between mild, moderate, severe, and very severe RLS. Optimal cut-off values of laboratory measurements were determined by Youden’s Index, i.e., the value corresponding to maximum (Sensitivity + 1 Specificity − 1). Evaluation of diagnostic validities for the cut-off values was reported with sensitivity and specificity with their 95% CIs. Analyses were performed with R v.3.6.3 (R Foundation for Statistical Computing, Vienna, Austria) statistical programming language. “coin” (v1.4-2) and “reportROC” (v3.6) libraries were used for nonparametric and diagnostic validity analyses, respectively. *p* < 0.05 was considered statistically significant.

## 3. Results

**Figure 2:**
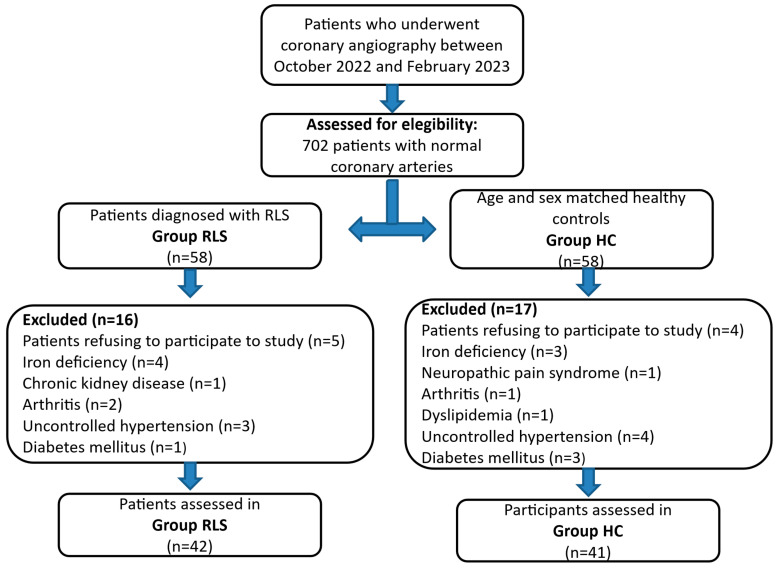
Study flow diagram.

A total of 116 participants were initially recruited for the study, with 58 individuals in the RLS group and 58 in the HC group. Ultimately, 83 participants (42 in the RLS group and 41 in the HC group) were included for analysis (Figure 2). The demographic and clinical characteristics of the participants are summarized in Table 1. Gender distribution, age, BMI, SAP, and HR were comparable between the groups (*p* > 0.05). Diastolic arterial pressure values were higher in RLS patients compared to HC (76.34 ± 7.91 vs. 79.4 ± 8.35, *p* = 0.047). The average disease severity score based on IRLSSG criteria was 16.54 ± 7.14 (range: 5–29).

**Figure 3:**
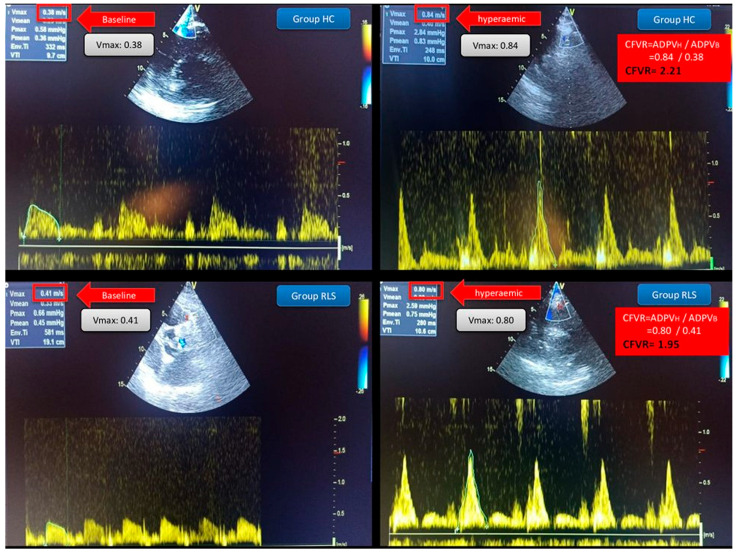
Average diastolic peak flow velocity values measured at baseline (ADPV_B_) and under hyperemic conditions (ADPV_H_) of one patient from each of the two groups. The coronary flow velocity reserve is defined as the ratio of ADPV_H_ to ADPV_B_. A CFVR value < 2.0 is considered abnormal.

Average diastolic peak flow values measured at baseline and under hyperemic conditions of one patient from each of the two groups are shown in Figure 3. In the RLS group, CFVR and ADPVH values were lower than those in the HC group (*p* < 0.001 for all). However, baseline ADPV values were similar between the groups (Table 2). All HC group participants had CFVR values above the normal range (CFVR ≥ 2.0), whereas 14 RLS patients had CFVR values below 2.0. The difference in the number of patients with CFVR values less than 2.0 between the groups was statistically significant (*p* < 0.001, 0/41 in HC group and 14/42 in RLS group).

**Figure 4:**
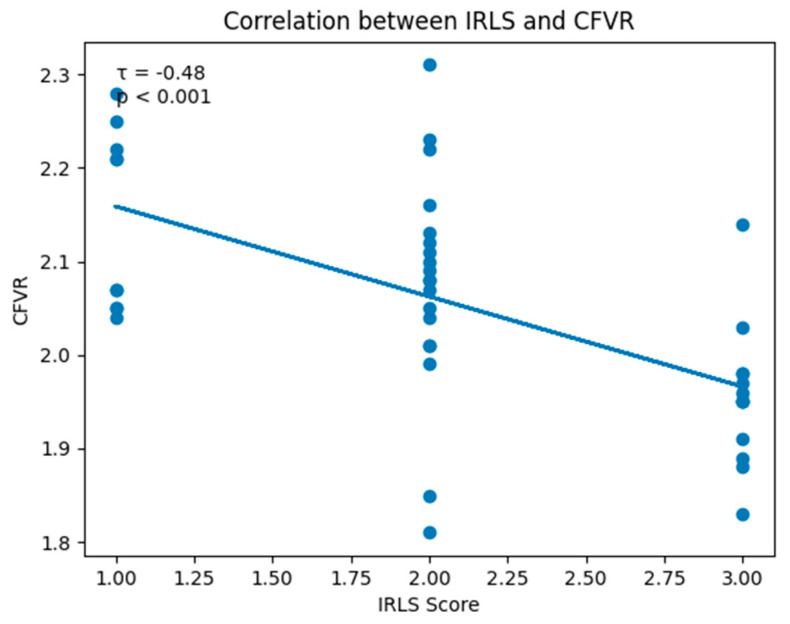
Scatterplot demonstrating the relationship between International Restless Legs Syndrome Study Group (IRLS) score and coronary flow velocity reserve (CFVR). Each point represents an individual participant. The fitted linear regression line illustrates a significant negative correlation between IRLS and CFVR (Kendall’s τ = −0.48, *p* < 0.001).

Patients with RLS exhibited higher WBC and CRP values compared to healthy controls, while other laboratory parameters showed no significant differences between the groups (Table 3). A statistically significant negative correlation was observed between CFVR and IRLS (*p* < 0.001, tau_b = −0.48) (Figure 4). However, the relationship between WBC, CRP, and IRLS was not statistically significant (*p* = 0.691, tau_b = −0.05; *p* = 0.279, tau_b = 137, respectively).

## 4. Discussion

This study represents the first investigation into the relationship between RLS and CFVR, building upon recent research linking RLS with CHD [9]. While previous studies have hinted at a connection between RLS and cardiovascular health, the direct correlation between CFVR and RLS severity had not been explored until now. Our findings reveal that individuals with RLS exhibit lower CFVR values compared to healthy controls, with a higher prevalence of abnormal CFVR values in the RLS group. Notably, as the severity of RLS increased, CFVR values decreased. Additionally, RLS patients displayed elevated WBC and CRP levels.

Coronary endothelial dysfunction serves as an independent predictor of acute cardiovascular events, regardless of angiographically detectable lesions [10,11]. Atherosclerosis begins early in life, and endothelial dysfunction contributes to this process at every phase of atherosclerosis [12]. Recently, several non-invasive methods for the assessment of endothelial function were introduced. The forearm flow-mediated vasodilatation (FMD) is one of these, and its measures correlate well with coronary endothelial function [13]. However, CFVR reflects global coronary microvascular and endothelial function and may serve as an indirect indicator of atherosclerotic burden, although it is not a specific marker of endothelial function per se [14]. Therefore, we preferred CFVR measurement to evaluate the relationship between RLS and coronary endothelial dysfunction in the current study. Recently, it was reported that the FMD was significantly lower in the patients with RLS when compared with healthy controls [15]. This has been interpreted as RLS being associated with endothelial dysfunction and therefore cardiovascular diseases. Similarly, Kim MS et al. reported that peripheral vascular endothelial functions are significantly impaired in RLS [16]. On the other hand, in a clinical study, RLS patients were found to have lower serum endocan levels, which is the marker of endothelial dysfunction. Authors suggest that patients with RLS may have better endothelial functions and that these patients may be better protected against atherosclerosis [17]. Moreover, the protective effects of RLS against atherosclerosis have been demonstrated in another study [18]. In the current study, we found that patients with RLS had lower CFVR, suggesting impaired coronary microvascular function, which may in part reflect underlying endothelial dysfunction. The reason for these different results in the literature may be related to the fact that the patients included in these studies were different in terms of gender and age distribution.

Restless leg syndrome may induce transient increases in heart rate and mean arterial pressure, and this sympathetic hyperactivity could lead to endothelial dysfunction and increase the risk for cardiovascular disease. Concerning the cardiovascular risk, while some studies have demonstrated an increased cardiovascular risk in RLS patients [19,20], others have found no such association [4,6]. Aksoy D et al. reported that patients with obstructive CHD have higher RLS prevalence. They have found that RLS was 3.05 times higher in patients with obstructive CHD than the patients with coronary arterial stenosis of less than 50%. However, in an article by Szentkirályi et al., it was determined that the presence of RLS was not a risk factor for cardiovascular morbidity [21]. We found that endothelial dysfunction, which is an early indicator of CHD, is more common in RLS patients and there is a negative correlation between disease severity and CFVR, indicating worsening endothelial function with increasing RLS severity. In addition to autonomic and inflammatory mechanisms, dopaminergic dysfunction, which plays a central role in the pathophysiology of RLS, may also contribute to vascular alterations. Reduced dopaminergic activity has been associated with increased sympathetic tone and impaired endothelial function. Moreover, dopamine has modulatory effects on vascular tone and inflammatory pathways, and its deficiency may promote endothelial dysfunction and low-grade inflammation. These mechanisms may partly explain the observed reduction in CFVR in patients with RLS; however, this relationship remains speculative and warrants further investigation.

In the literature, it was reported that a CFVR < 2.0 is considered abnormal, and lower CFVR indicates coronary endothelial dysfunction [9]. According to our results, the mean CFVR values of the patients in the group RLS were 2.06. Although the mean CFVR value in patients with RLS was statistically lower than in healthy controls, the mean value of CFVR in this group was above the normal range. This may be related to the fact that none of the patients included in the current study had very severe RLS. On the other hand, the number of participants with a CFVR value below 2 was significantly higher in the RLS group. While there were no patients in the control group with a CFVR value below 2.0, 14 patients in the RLS group had a CFVR lower than 2.0. Moreover, there was a negative correlation between IRLS and CFVR. These findings suggest that there is a non-negligible relationship between RLS and coronary endothelial dysfunction. Our findings of elevated WBC and CRP levels in RLS patients are consistent with previous studies reporting increased inflammatory markers such as hsCRP and interleukin-6 in this population, supporting the role of systemic inflammation in RLS pathophysiology [19]. However, the absence of a statistically significant correlation between these inflammatory markers and CFVR in our study suggests that systemic inflammation, as measured by WBC and CRP, may not be the primary driver of microvascular dysfunction in RLS. This discordance could reflect the insensitivity of these non-specific markers in capturing the local vascular inflammatory milieu, or alternatively, that the microvascular impairment observed in RLS is mediated predominantly through autonomic or dopaminergic mechanisms rather than systemic inflammation. These findings should therefore be interpreted cautiously, and the proposed mechanistic link between inflammation and reduced CFVR in RLS remains to be established.

A higher prevalence of RLS in patients with systemic inflammatory diseases might demonstrate the role of inflammation in RLS pathophysiology [22]. Trotti LM. reported that serum levels of high-sensitivity C-reactive protein (hsCRP), interleukin-6 (IL-6), ferritin, and N-terminal pro-B type natriuretic peptide were found to be higher in patients with RLS compared to the healthy controls [23]. In line with the literature, we found higher WBC and CRP values in patients with RLS. All these results show that there may be a close relationship between the systemic inflammatory response and RLS.

A further limitation of the present study is the absence of objective sleep assessment. Obstructive sleep apnea (OSA) is highly prevalent among patients with RLS and is independently associated with impaired CFVR and endothelial dysfunction. Since polysomnography or validated screening tools (e.g., STOP-BANG questionnaire) were not performed, the possibility that unrecognized coexisting sleep-disordered breathing contributed to the observed reduction in CFVR cannot be excluded. Future studies should incorporate objective sleep assessment to disentangle the independent contributions of RLS and OSA to coronary microvascular function.

The present study has the following limitations. First, our modest sample size obtained from a single center may limit the generalizability of the observed results. No formal power calculation was performed prior to the study, and the sample size may have been insufficient for stable ROC-derived cut-off determination. The diagnostic thresholds reported should therefore be interpreted with caution and validated in larger cohorts. Second, an assessment of additional markers of endothelial dysfunction besides CFVR, which could have supported the study results, was not performed. Additionally, more specific endothelial and inflammatory biomarkers, such as nitric oxide-related parameters and interleukin-6, were not assessed, which could have provided further mechanistic insight. Third, none of the patients included in our study had very severe RLS. The inclusion of very severe RLS patients in the current study could have changed our results. Fourth, the absence of a long-term follow-up of the participants for CHD. Although all participants tested negative for active COVID-19 infection by PCR at the time of evaluation, the potential impact of prior asymptomatic or undocumented COVID-19 infection on inflammatory markers and endothelial function cannot be completely excluded. Therefore, residual confounding related to the pandemic period should be considered when interpreting the results. In addition, although several clinical variables were recorded, potential confounders such as smoking status, medication use, sleep-related disorders, and other inflammatory conditions were not fully adjusted for in the analysis, which may have influenced the observed associations. Due to the relatively small sample size, performing a fully adjusted multivariable regression model may have resulted in model overfitting. Therefore, the current analysis was primarily designed as an exploratory assessment of the association between RLS severity and CFVR. Lifestyle-related factors, including physical activity level and sedentary behavior, were not evaluated and may also have influenced the results. Although lifestyle-related factors such as physical activity level were not directly assessed, BMI values were comparable between groups, which may indirectly suggest a similar baseline metabolic profile. In addition, participants were selected from patients undergoing coronary angiography for clinical indications, which may introduce selection bias and limit the generalizability of the findings to the broader RLS population. Patients referred for coronary angiography may represent a higher-risk cardiovascular subgroup compared with community-dwelling individuals with RLS, potentially overestimating the prevalence of microvascular dysfunction in this population. However, this recruitment strategy also ensured that all individuals had objectively confirmed normal epicardial coronary arteries, which is an important methodological strength, as it allows a more accurate and isolated assessment of microvascular function without the confounding effect of obstructive coronary artery disease. Future studies enrolling patients with established RLS across a wider severity spectrum—including very severe cases—and subjecting them to the same diagnostic protocol regardless of clinical indication may provide a more representative picture of the relationship between RLS and coronary endothelial dysfunction.

## 5. Conclusions

We conclude that the patients with RLS had lower CFVR values compared with healthy controls and there was a negative correlation between the severity of RLS and CFVR which indicates coronary endothelial function. These exploratory findings suggest a potential association between RLS and impaired coronary microvascular function, which may warrant further investigation in prospective studies with longer follow-up. Whether routine cardiovascular surveillance is beneficial in RLS patients, particularly those with severe disease, remains to be determined in future outcome-based research. However, given the exploratory and cross-sectional nature of this study and the absence of multivariable adjustment, these findings should be interpreted with caution and require confirmation in future prospective studies.

## Figures and Tables

**Table 1:** Demographic and clinical characteristics of the participants.

	All (n = 83)	Group HC (n = 41)	Group RLS (n = 42)	*p*
Gender F/M (n/%)	44 (53)/39 (47)	21 (51.2)/23 (54.8)	20 (48.8)/19 (45.2)	0.746 *
Age	47.08 ± 6.84 47 (34–69)	47.83 ± 7.49 48 (36–69)	46.36 ± 6.14 46.5 (34–63)	0.330 ^†^
BMI	29.54 ± 3.14 29.3 (24.4–35.5)	29.29 ± 3 28.8 (24.7–34.7)	29.79 ± 3.28 29.45 (24.4–35.5)	0.509 ^‡^
SAP	125.06 ± 10.4 125 (95–150)	123.17 ± 8.93 125 (100–145)	126.9 ± 11.47 130 (95–150)	0.102 ^†^
DAP	77.89 ± 8.23 80 (60–100)	76.34 ± 7.91 80 (60–90)	79.4 ± 8.35 80 (60–100)	0.047 ^‡^
HR	78.18 ± 11.33 78 (56–104)	79 ± 10.27 76 (63–103)	77.38 ± 12.35 78 (56–104)	0.518 ^†^

Numerical data are summarized as mean ± standard deviation along with median (minimum–maximum), whereas frequency and percentage, n (%), were used for categorical data. *p* values are obtained by Student’s *t*-test (^†^), Mann–Whitney U test (^‡^) and chi-square test (*). *p* < 0.05 is accepted as statistically significant. Abbreviations: BMI, body mass index; DAP, diastolic arterial pressure; Group HC, healthy control group; Group RLS, restless leg syndrome group; HR, heart rate; and SAP, systolic arterial pressure.

**Table 2:** Comparison of groups in terms of CFVR, ADPV_B_, and ADPV_H_ values.

	All (*n* = 83)	Group HC (*n* = 41)	Group RLS (*n* = 42)	*p*
CFVR	2.21 ± 0.19 2.23 (1.81–2.53)	2.37 ± 0.08 2.39 (2.16–2.53)	2.06 ± 0.12 2.05 (1.81–2.31)	<0.001
ADPV_B_	34.8 ± 2.78 34.8 (28.3–40.8)	35.36 ± 3.15 35.6 (28.3–40.8)	34.25 ± 2.29 33.9 (29.8–39.2)	0.071
ADPV_H_	76.99 ± 9.53 76.4 (61.3–97.1)	83.77 ± 7.84 85.3 (66.3–97.1)	70.37 ± 5.56 68.91 (61.3–81.2)	<0.001

Numerical data are summarized as mean ± standard deviation along with median (minimum–maximum). *p* values are obtained by Student’s *t*-test. *p* < 0.05 is accepted as statistically significant. Abbreviations: ADPV_B_, average diastolic peak flow velocity measured at baseline; ADPV_H_, average diastolic peak flow velocity measured under hyperemic conditions; CFVR, coronary flow velocity reserve; Group HC, healthy control group; and Group RLS, restless leg syndrome group.

**Table 3:** Comparison of groups in terms of laboratory parameters.

	All (*n* = 83)	Group HC (*n* = 41)	Group RLS (*n* = 42)	*p*
WBC	6.79 ± 2.34 6 (3.6−14.5)	5.91 ± 1.55 5.5 (3.6−10.4)	7.64 ± 2.66 6.8 (3.9−14.5)	0.001 *
CRP	0.84 ± 0.43 0.8 (0.1–2.2)	0.73 ± 0.38 0.6 (0.1−1.9)	0.94 ± 0.44 0.95 (0.3−2.2)	0.02 *
Glucose	96.25 ± 9.69 96 (76−122)	95.41 ± 10.51 95 (76−121)	97.07 ± 8.86 97 (79−122)	0.439 ^†^
HDL	53.89 ± 14.58 54 (26−88)	56.34 ± 14.89 58 (29−88)	51.5 ± 14.04 50.5 (26−79)	0.131 ^†^
LDL	143.28 ± 43.67 144 (67−278)	146.68 ± 42.31 145 (67−256)	139.95 ± 45.22 138 (69−278)	0.486 ^†^
Creatinine	1.03 ± 0.23 1.1 (0.6−1.4)	1.04 ± 0.23 1.1 (0.6−1.4)	1.02 ± 0.22 1.1 (0.6−1.4)	0.726 *
Hbg	13.79 ± 1.63 13.6 (10.5−18.3)	13.86 ± 1.64 13.6 (10.5−18.3)	13.72 ± 1.65 13.65 (11.1−18.3)	0.538 *

Numerical data are summarized as mean ± standard deviation along with median (minimum–maximum). *p* values are obtained by Student’s *t*-test (^†^) and Mann–Whitney U test (*). *p* < 0.05 is accepted as statistically significant. Abbreviations: CRP, C-reactive protein; Group HC, healthy control group; Group RLS, restless leg syndrome group; Hbg, hemoglobin; HDL, high-density lipoprotein cholesterol; and LDL, low-density lipoprotein cholesterol.

## Data Availability

The datasets generated and/or analyzed during the current study are available from the corresponding author upon reasonable request.

## Abbreviations

RLS	Restless legs syndrome
CFVR	Coronary flow velocity reserve
IRLS	International Restless Legs Scale
WBC	White blood cell count
CRP	C-reactive protein
HDL	High-density lipoprotein
LDL	Low-density lipoprotein
BMI	Body mass index
SAP	Systolic arterial pressure
DAP	Diastolic arterial pressure
HR	Heart rate
ADPV	Average diastolic peak flow velocity
ADPVB	Average diastolic peak flow velocity at baseline
ADPVH	Average diastolic peak flow velocity under hyperemic conditions
ROC	Receiver operating characteristic
AUC	Area under the curve
CI	Confidence interval
